# Do the Gender and the Number of Comorbidities and the Use of Tertiary Prevention Play a Role in the Severity of Anxiety and Depression in Patients with Coronary Artery Disease? A POLASPIRE II Study

**DOI:** 10.3390/jcm13133812

**Published:** 2024-06-28

**Authors:** Józefa Dąbek, Zbigniew Gąsior, Marek Styczkiewicz, Aldona Kubica, Dariusz A. Kosior, Renata Wolfshaut-Wolak, Marek Rajzer, Magdalena Szynal, Piotr Jankowski, Karol Kamiński

**Affiliations:** 1 Department of Cardiology, Faculty of Health Sciences, Medical University of Silesia in Katowice, Ziołowa Street 45-47, 40-635 Katowice, Poland; jdabek@sum.edu.pl (J.D.); zgasior@sum.edu.pl (Z.G.); magdalena.szynal@sum.edu.pl (M.S.); 2 Department of Cardiology, Independent Public Provincial Hospital, Jana Pawła II 10, 43-170 Zamość, Poland; styczkiewicz@interia.pl; 3 Department of Cardiac Rehabilitation and Health Promotion, Nicolaus Copernicus University in Torun, Collegium Medicum in Bydgoszcz, Skłodowskiej-Curie 9, 85-094 Bydgoszcz, Poland; aldona.kubica@gmail.com; 4 Mossakowski Medical Research Institute, Polish Academy of Sciences, Adolfa Pawińskiego 5, 02-106 Warsaw, Poland; dkosior13@gmail.com; 5 Institute of Nursing and Midwifery, Jagiellonian University Medical College, Michałowskiego 12, 31-008 Krakow, Poland; rwolfshaut@gmail.com; 6 Institute of Cardiology Collegium Medicum, Jagiellonian University, 31-008 Krakow, Poland; rajzer37@interia.pl; 7 Department of Internal Medicine and Gerontology, Medical Center for Postgraduate Education, 01-813 Warsaw, Poland; piotrjankowski.eu@gmail.com; 8 Department of Population Medicine, Medical University Bialystok, M. Skłodowskiej-Curie 24A, 15-089 Bialystok, Poland

**Keywords:** depression, anxiety, coronary heart disease, POLASPIRE

## Abstract

**Background/Objectives:** The need to conduct research on anxiety and depression in patients with coronary artery disease in connection with factors such as gender or implemented tertiary prevention is very important for drawing practical conclusions and, consequently, implementing new recommendations and procedures. The aim of the study was to attempt to answer the question whether gender and the number of comorbidities, as well as the application of tertiary prevention principles, play a role in the severity of anxiety and depression in the studied group of patients with coronary artery disease. **Material:** The study involved 765 patients from 11 Polish cardiology centers. The presented material is part of the multicenter POLASPIRE II study. **Methods**: All patients completed The Hospital Anxiety and Depression Scale (HADS) questionnaire, and a medical interview was conducted with them. **Conclusions:** Although the intensity of anxiety and depression in the studied group of patients was low, gender differentiated them, which, however, did not influence undertaking tertiary prevention activities. In the study group of patients, the number of comorbidities and cardiac incidents/procedures after the event qualifying for the study, as well as preventive actions undertaken, were not associated with the severity of anxiety and depression. In the studied group of patients with coronary heart disease, there was still a large group of people who did not take preventive measures. Therefore, there is a need for systematic education regarding the benefits of implementing them to prevent the progression of the disease and premature death.

## 1. Introduction

According to various sources, it is estimated that between 280 and even 350 million people suffer from depression in the world [1,2]. In patients with coronary artery disease, the incidence of major depressive disorder (MDD—according to the DSM-5 classification) is estimated at 15–20%, while symptoms indicating mild depressive disorders during hospitalization due to myocardial infarction or during the following year present up to 45% [3]. Patients with coronary artery disease are also three times more likely to develop these disorders compared to the general population [4,5].

There are also reports that depression occurs in people with heart failure and peripheral arterial disease. Among the Polish population, depressive symptoms occurred much more often in people with hypertension, coronary heart disease, stroke, and diabetes [4,5].

The presence of depressive symptoms, compared to people without symptoms of depression, causes increased mortality from cardiac causes, as well as increased overall mortality, regardless of the severity of the somatic disease [5].

According to the literature, the pathophysiological mechanisms linking depression and ischemic heart disease include disturbances in the functioning of the hypothalamic–pituitary–adrenal axis, disturbances in the equilibrium between the sympathetic and parasympathetic systems, abnormal functioning of the coagulation system, chronic inflammation, and dysfunction of the endothelium of blood and lymphatic vessels [5].

Stress and emotional factors (including anxiety and depression) affect the function of the hypothalamic–pituitary–adrenal axis and the activation of the sympathetic nervous system. In response to a stress stimulus, the release of the adrenocorticotropic hormone increases, thereby stimulating the release of corticosteroids in the adrenal cortex (cortisol) and their elevated concentration in the blood. Chronically elevated cortisol levels lead to the development of, among others, cardiovascular diseases [5]. Cortisol increases the vasoconstricting effects of adrenaline, which can lead to hypertension, a risk factor for coronary heart disease [6]. Furthermore, it has a direct impact on many factors involved in the pathogenesis of arteriosclerosis by chronically increasing the levels of cholesterol, glucose, and fatty acids.

In the pathogenesis of coronary artery disease, atherosclerotic plaque is formed on the basis of inflammation, with an accompanying increase in pro-inflammatory cytokines. Moreover, published works also reveal increased concentrations of pro-inflammatory cytokines accompanying the development of depression. Coronary heart disease is often accompanied by health-related anxiety and depression. Studies have shown that the stimulation of cytokine synthesis or the administration of pro-inflammatory cytokines leads to the development and intensification of depressive symptoms, while the use of antidepressant treatment reduces the concentration of cytokines in depressed patients [5].

Anxiety is the feeling of fear that occurs when faced with threatening or stressful situations [7]. Research shows a connection between anxiety and cardiovascular diseases, especially in older people [8]. Pathological anxiety has been implicated in the development of other medical problems such as alcoholism, psychoactive substances, and cigarette abuse, which are risk factors for the development of cardiovascular diseases [9]. According to the studies, 32% of patients with heart failure (HF) experience elevated levels of anxiety, and 13% meet criteria for an anxiety disorder. Moreover, 25% of patients after cardiac artery bypass grafting (CABG) experience anxiety [10]. A meta-analysis including 20 studies and nearly 250,000 patients found that anxiety, controlling for other medical variables, when possible, led to a 26% increased risk of incident of coronary artery disease (CAD) [11]. Moreover, in patients with anxiety disorders, there is a significant reduction in high-frequency heart rate variability, which is the domain of parasympathetic control of the heart rate. A more pronounced increase in heart rate inevitably results in a greater myocardial demand for oxygen, which may result in ischemia [12].

Prevention in patients with coronary heart disease involves changing lifestyle with the elimination of modifiable risk factors, such as improper nutrition, overweight and obesity, immoderate alcohol consumption, smoking, little or no physical activity, and hypertension and diabetes. Such actions help prevent the progression of coronary heart disease and, consequently, repeated heart attacks and death. The occurrence of depression in patients with cardiovascular diseases may significantly reduce their motivation to implement the above-mentioned preventive measures and, as a result, lead to frequent repeated coronary events [13].

The aim of the study was to attempt to answer the question whether gender and the number of comorbidities, as well as the application of tertiary prevention principles, play a role in the severity of anxiety and depression in the studied group of patients with coronary artery disease.

## 2. Materials and Methods

The study involved 765 patients from 11 Polish cardiology centers. Patients qualified for the study if they had been hospitalized in the last 6 to 18 months due to myocardial infarction (with or without ST-segment elevation), unstable angina, percutaneous coronary interventions (PCIs), or qualification for surgical treatment of coronary artery disease (cardiac artery bypass grafting—CABG).

The study was initiated after obtaining the consent of the Bioethics Committee of Medical University of Silesia (no. PCN/CBN/0022/KB1/97/21), and all patients gave informed consent to participate in the study. The presented material is part of the multicenter POLASPIRE II study.

All patients included in the study completed the HADS questionnaire on their own. The Hospital Anxiety and Depression Scale (HADS) is used to assess anxiety and depression in patients with various diseases. The questionnaire contains two independent subscales for assessing anxiety and depression, each of which consists of seven statements. The answers are constructed according to a 4-point Likert scale, scored from 0 to 3. The final score for each subscale ranges from 0 to 21 points. A correct result is marked from 0 to 7 points; from 8 to 10 means a borderline result, while from 11 to 21 indicates a clinically significant result [14,15].

In addition, a thorough medical interview was conducted with each patient, during which the above-mentioned questionnaire was supplemented with questions about comorbidities and principles of tertiary prevention of coronary heart disease.

## 3. Statistical Analysis

Statistical analyses were performed using Statistica ver. 13.1 (Statsoft, Kraków Poland). The results of the study group were prepared by presenting qualitative data, such as the number of respondents in individual groups and percentages in relation to the entire population and quantitative data considering descriptive statistics, i.e., mean, median, standard, and quartile deviations. The Mann–Whitney U test was used to calculate the differences between the two compared groups. Spearman’s rank test and the Chi 2 test were used for correlation analysis. In all analyses performed, the level of statistical significance was set at *p* < 0.05. Furthermore, to check the potential impact of many variables on the number of points obtained in the questionnaire, the multiple regression method was used.

## 4. Results

Table 1 shows the general characteristics of the study group. Almost three-quarters of the study group were men (573; 74.37%). Most people were aged 60–71 (312; 40.78%). The average age was 66.67 ± 8.51. Patients were most often qualified for the study based on a history of myocardial infarction without ST-segment elevation (197; 25.75%).

Table 2 presents the characteristics of the study group, including the number of selected health incidents or procedures after an event qualifying for the study. After the event qualifying for the study, patients most often reported percutaneous coronary angioplasty as a repeat procedure/health incident (95; 12.42%).

Table 3 presents the characteristics of the study group, including the number of tertiary prevention activities undertaken after the event qualifying for the study. In terms of tertiary prevention, the surveyed patients most often monitored their blood pressure (660; 86.27%), and in terms of the number of measures implemented, they most often declared four of them (184; 24.05%).

Table 4 presents the characteristics of the study group, including the number of comorbidities. Most patients had hypertension (584; 76.34%) and hypercholesterolemia (583; 77.21%), and the presence of two comorbidities was declared by almost one-third of the respondents (147; 32.29%).

Table 5 presents the assessment of the severity of depression and anxiety of the examined patients, considering the gender. The severity of anxiety and depression among both women (M = 6.63; M = 5.05) and men (M = 4.96; M = 4.29) was within the normal range. The median anxiety and depression severity scores in the study group were higher in women than in men (7.00 vs. 4.00; *p* = 0.006 and 4.50 vs. 4.00; *p* < 0.001, respectively).

Table 6 presents the characteristics of the study group, including the analysis of the relationship between the assessment of the severity of depression and anxiety and the number of comorbidities, the number of preventive actions taken, and the number of incidents/treatments after the event qualifying for the study. The result of none of the above correlations was statistically significant, which indicates that the analyzed variables are not associated with the severity of depression and anxiety.

Table 7 presents the characteristics of the study group, including the relationship between gender and the number of preventive actions taken. The correlation between gender and the number of preventive actions undertaken by the respondents was not statistically significant, which indicates that in the study group, gender was not related to the number of tertiary prevention actions implemented.

Moreover, a regression analysis was performed with all examined factors, but only gender had a significant effect, as shown in Table 8. The prepared models explained only 3.4% and 2.3% of the variability of scores assessing the severity of drug and depression among the studied patients. However, it was shown that in both cases, male gender had a significant impact on the number of points obtained.

## 5. Discussion

Among the comorbidities, most respondents had hypertension (584; 76.34%), and in terms of tertiary prevention, they most often declared monitoring of blood pressure (660; 86.27%). The study by T. Keating et al. analyzed compliance with recommendations regarding prevention in patients after CABG. Only slightly more than half of the examined patients (92; 53.2%) declared that their blood pressure was regularly monitored and was within the normal range, while 16 (9.2%) reported elevated blood pressure values [16]. R. Sudevan et al., in their study on compliance with measures taken by patients in India to prevent coronary heart disease, showed that blood pressure was controlled by 65.11% of the respondents. In the above study, as many as 93.86% of people stopped smoking [17]. In our study, only 380 (49.67%) patients quit smoking, while in the study by T. Keating et al., 108 (62.4%) quit [16]. K. Kotseva et al. collected data from 27 countries, including on risk factors for coronary heart disease. They showed that 55% of respondents smoked cigarettes and 53% declared quitting smoking in the 6 months that followed [18]. In the research of R.D. Santos, 50% of smokers still had not quit smoking, and only 5% of respondents went to a smoking cessation clinic. This was despite these services being offered to 85% of people who still smoked [19]. In the Kotseva et al. analysis, 78% of patients were confirmed to be taking medications to lower blood pressure. The percentage of people with blood pressure 130/80 mmHg was 71%, while 42% had blood pressure 140/90 (140/85 mmHg for diabetes) and 12% had 160/100 mmHg [18].

In our study, 261 (34.11%) patients regularly monitored their glycemia, while in the analysis conducted by R. Sudevan et al. it was 51.23% [17]. In the study by T. Keating et al., 102 (59%) respondents declared their cholesterol to be normal, and 13 (7.5%) declared it to be high, despite the pharmacotherapy used. Just over half (98; 56.6%) followed a healthy diet “most of the time” [16]. In turn, in our study, as many as 583 (76.21%) people had hypercholesterolemia and only 154 (20.13%) followed a cholesterol-lowering diet. In the analysis of K. Kotseva et al., the proportion of patients with an LDL-C level of 1.8 mmol/L (70 mg/dL) was 71% and 37% of all patients had an LDL-C level of 2.5 mmol/L (100 mg/dL). They also showed that 44% of patients were overweight, 38% of patients were obese, and about 40% of patients did not receive any dietary guidelines and, therefore, did not follow a diet [18]. R.D. Santos in his analysis observed an increase in obese people from 25% to 38% in the EUROASPIRE I and IV/V registries. He also emphasized that cholesterol is apparently less controlled in EUROASPIRE IV and V than in previous studies [19].

As research has shown, the nutrition of Poles is far from proper. The National Institute of Public Health—National Institute of Hygiene (NIZP-PZH) published a report that revealed that the amount of bread and flour eaten by Poles decreased by over 30%; the consumption of potatoes declined by 35% and that of other vegetables by 6%, and the consumption of fruit increased by 6%. Poles also increased their consumption of red meat by as much as 120%. According to the World Cancer Research Fund (WCRF) and the American Institute for Cancer Research (AICR), the consumption of red meat for an adult should not exceed 71 g, and the average Pole ate from 120 to 129 g of red meat and various meat products daily. Unfortunately, the consumption of fish also decreased by as much as 40% and the number of confectionery products eaten by Poles, as well as crispbread, rice wafers, and other bakery products, increased by 20% [20].

According to the recommendations of the World Health Organization (WHO), adults should undertake moderate (150–300 min/week) or intense (75–150 min/week) exercise or an equivalent combination of moderate and intense exercise [21]. Data from the study “Level of Physical Activity of Poles 2018” conducted by the Ministry of Sport and Tourism showed that only 21.8% of Poles met WHO standards regarding the level of physical activity in their free time [22]. In our study, 264 (34.50%) declared regular physical activity. In the analysis by R. Sudevan et al., this figure was 39.22% [17]. However, in the study by T. Keating et al., 143 (82.7%) people regularly engaged in physical activity, at least 1–2 times a week, or even every day [16]. In the research performed by K. Kotseva et al., two-thirds (66%) of patients did not meet their stated physical activity goal and only 16% engaged in vigorous exercise [18].

R. Fernández Coronado and A. Olórtegui Yzu conducted a study on the effectiveness of prevention in patients with coronary artery disease in Peru. They showed that it was associated with a better quality of life, and an improvement in parameters such as body weight, abdominal circumference, body mass index (BMI), and the physical capacity of the examined patients was observed. However, they did not show any changes in the patient’s lipid profile (except for an increase in the HDL fraction) or glycemia [23]. In their study on measures taken by patients in China to prevent coronary heart disease, M. Lu et al. showed that the subjects only complied with the recommendations regarding regular medication intake, while their prevention in terms of changing their lifestyle was not sufficient and required improvement [24].

Our study assessed the severity of anxiety and depression. Both among women (M = 6.63; M = 5.05) and men (M = 4.96; M = 4.29), the values were within the normal range. Despite this, the median anxiety and depression severity scores were higher in women than in men (7.00 vs. 4.00; *p* = 0.006 and 4.50 vs. 4.00; *p* < 0.001, respectively).

Many studies indicate a higher incidence of depression in women in various population groups [25,26,27,28]. This was demonstrated, among others, by S. Mohammadi et al. in their analysis of gender differences in the development of depression, as well as by R. Thériault and M. Perreault in their studies on hormonal regulation in connection with, among others, gender and depression [25,26].

“Female” depression most often manifests itself differently than “male” depression. Women with depression are more likely to have impaired appetite and sleep, depressed mood, and fatigue, which more closely reflects the diagnostic criteria for MDD. However, in men, depression is much more often manifested by anger attacks, aggression, use of psychoactive substances, and risky behaviors [25,26]. However, men are more than three times more likely to die from depression-related suicides than women [27].

M. Komendarek-Kowalska analyzed a group of cancer patients using the standardized HADS questionnaire. She showed a relationship between the occurrence of depressive symptoms and anxiety disorders and gender (the above-mentioned ailments occurred much more often in women than in men) [28]. Also, A. Wójcikowska, who examined the quality of life and the frequency of depressive symptoms among patients with chronic obstructive pulmonary disease using the Beck Depression Scale, showed a more frequent occurrence of depressive symptoms in women [29].

The reasons for the discussed difference in the greater susceptibility to depression of women than men are seen in many social factors, such as gender inequality, unfair wages, exclusion on the labor market, burden of household duties, lack of help from a partner, greater social “permission” in their life, a feeling of “failure” in their life compared to men, and in changes in women’s hormonal balance (e.g., pregnancy and menopause) [25,26].

R. Piotrkowska et al. used the HADS questionnaire on 62 patients with peripheral artery disease (PAD) treated at the Department of Cardiac Surgery and Vascular Surgery of the University Clinical Center in Gdańsk. They showed that the level of depression in patients increased as their pain increased [30]. In her analyses, A. Wójcikowska also observed a relationship between the advancement of the disease and the severity of depressive symptoms [29]. B. Tang et al. conducted a study on the occurrence of depression among patients with cardiac and metabolic diseases. They showed a strong association of depressive symptoms with type 2 diabetes and coronary heart disease, as well as a slightly significant association between MDD and heart failure [31]. W. Akosile et al., in their studies on the treatment of depression in patients with coronary artery disease, found that the occurrence of depressive symptoms may be considered a potential cardiovascular risk factor [32].

M. Açıkel, in his research on depression and anxiety among patients undergoing CABG surgery, showed that the level of both depression and anxiety was significantly higher after the procedure than before it, while the correlations with age, gender, and profession were not statistically significant [33]. M.T. Bekendam et al. demonstrated an association between anxiety and the severity of myocardial ischemia in relation to two demographic factors, gender and age. In this analysis, anxiety was associated with severe myocardial ischemia on myocardial perfusion single-photon emission computed tomography (SPECT) and it was significant only for women ≤ 65 years old [34]. G. Wang et al. investigated the impact of anxiety on the prognosis of coronary artery disease in patients of Chinese Han ethnicity. They showed that anxiety was independently associated with the severity of coronary atherosclerosis and predicted a worse outcome in patients with CAD [35]. S.H. Abbasi et al. investigated factors associated with anxiety in premature coronary artery disease patients. Through a regression analysis, they showed different significant factors associated with anxiety in men and women. In the male group, these were opium use, positive family history of CAD, and high creatinine levels. In women, these were major adverse cardiac events (MACEs) during follow-up, hypertension, and the duration of CAD [36]. P. Vynckier et al. also observed a relationship between gender and higher levels of anxiety and depression among women. Their analysis also showed that the more comorbidities patients had, the worse the results mentioned [37]. A. Pająk et al. showed that women and those patients not undergoing invasive treatment appear to be more susceptible to depression and anxiety [38].

Our study analyzed the correlations of the severity of anxiety and depression with the number of preventive actions taken, the number of cardiac incidents/procedures after an event qualifying for the study, and the number of comorbidities. The result of none of the above-mentioned correlations was statistically significant, suggesting that the variables analyzed above were not related to the assessment of the severity of depression and anxiety. The obtained results are probably this way because the HADS questionnaire was completed by the patients on the day of their examination and not at the time of hospitalization due to a cardiac event, based on which the patients were qualified for the study.

An analysis of the relationship between gender and the number of preventive actions undertaken was also performed, and the result was also not statistically significant, suggesting that in the study group, gender was not associated with the implementation of tertiary prevention.

The need to conduct research on anxiety and depression in patients with coronary artery disease in connection with factors such as gender or implemented tertiary prevention is still current to create databases that make it possible to compare changing relationships under the influence of various environmental factors, such as the COVID-19 pandemic and flu. A comparative analysis of the above-mentioned factors allows for the drawing of practical conclusions and, consequently, implementing new recommendations and procedures.

The study is not free from limitations. Tertiary prevention and its implementation by patients was based on respondents’ responses, not on medical records. Moreover, the study group included patients both 6 and 18 months after a coronary event with varying degrees of severity, so individual people may have had a greater tendency to adhere to tertiary prevention than others. Another limitation is that the HADS questionnaire was completed by the respondents on the day of the examination and not during their hospitalization.

## 6. Conclusions

Although the intensity of anxiety and depression in the studied group of patients was low, gender differentiated them, which, however, did not influence undertaking tertiary prevention activities. In the study group of patients, the number of comorbidities, cardiac incidents/procedures after the event qualifying for the study, and preventive actions undertaken were not associated with the severity of anxiety and depression. In the studied group of patients with coronary heart disease, there was still a large group of people who did not take preventive measures. Therefore, there is a need for systematic education regarding the benefits of implementing them to prevent the progression of the disease and premature death.

## Figures and Tables

**Table 1:** General characteristics of the study group.

Study Group (765; 100%)			
Variables		Data	
		n	%
**Sex**	Women	192	25.63
	Men	573	74.37
**Age** **(years)**	30–40	5	0.65
	41–50	36	4.71
	51–60	119	15.56
	61–70	312	40.78
	71–80	293	38.30
**An event qualifying for the study** **with ST-segment elevation**	Myocardial infarction without ST-segment elevation	197	25.75
	Myocardial infarction with ST-segment elevation	185	24.18
	Unstable angina	142	18.56
	Planned percutaneous coronary angioplasty	148	19.35
	Elective coronary artery bypass grafting	93	12.16

**Table 2:** Characteristics of the study group, including the number of selected incidents of health care and treatments after an event qualifying for the study.

Study Group (765; 100%)		
Variables	Data	
	n	%
**Health incidents and treatments after the event qualifying for the study**		
Percutaneous coronary angioplasty	95	12.42
Angina pectoris	38	4.97
Heart failure	35	4.57
Coronary artery bypass grafting	22	2.88
Myocardial infarction	20	2.61
COVID-19 infection	19	2.48
Peripheral arterial disease	8	1.04
Stroke	3	0.39
**Number of incidents and procedures**		
0	579	75.69
1	7	0.92
2	130	16.99
3	41	5.36
5	8	1.05

Explanation of abbreviations: n—number of participants.

**Table 3:** Characteristics of the study group, including the number of tertiary prevention activities undertaken after an event qualifying for the study.

Study Group (765; 100%)		
Variables	Data	
	n	%
**Tertiary prevention activities**		
Blood pressure monitoring	660	86.27
Taking medications for hypertension	566	73.99
Not drinking alcohol	484	63.28
Stopping smoking	380	49.67
Regular physical activity	264	34.50
Glycemic monitoring	261	34.11
Weight reduction	179	23.40
Cholesterol-lowering diet	154	20.13
Diet to lower blood pressure	151	19.74
**Number of tertiary prevention activities implemented**		
0	14	1.83
1	26	3.40
2	83	10.85
3	171	22.35
4	184	24.05
5	144	18.82
6	87	11.37
7	34	4.44
8	20	2.61
9	2	0.26

Explanation of abbreviations: n—number of participants.

**Table 4:** Characteristics of the study group, including comorbidities.

Study Group (765; 100%)		
Variables	Data	
	n	%
**Concomitant diseases**		
Hypertension	584	76.34
Hypercholesterolemia	583	76.21
Obesity	312	40.78
Diabetes	257	33.59
Kidney disease	96	12.55
Peripheral vascular disease	77	10.07
**Number of comorbidities**		
0	37	4.84
1	114	14.90
2	247	32.29
3	213	27.84
4	112	14.64
5	38	4.97
6	4	0.52

Explanation of abbreviations: n—number of participants.

**Table 5:** Assessment of the severity of depression and anxiety in the examined patients, considering gender.

Study Group (765; 100%)										
Variables	Sex	M	SD	Me	Min.	Max.	Q1	Q3	Z	*p*
**Assessment of the severity of depression**	**Women**	5.05	3.42	4.50	0	16.0	2.00	7.00	5.51	*p* < 0.001
	**Men**	4.29	3.31	4.00	0	14.0	2.00	7.00		
**Assessment of anxiety severity**	**Women**	6.63	3.77	7.00	0	17.0	3.00	9.00	2.73	*p* = 0.006
	**Men**	4.96	3.58	4.00	0	20.0	2.00	7.00		

Explanation of abbreviations: M—median, SD—standard deviation, Me—median, Min.—minimum value, Max.—maximum value, Q1—lower quartile, Q3—upper quartile, Z—the value of the Mann–Whitney U statistic for the two compared groups, *p*—the value of statistical significance.

**Table 6:** Characteristics of the study group, including the analysis of the relationship between the assessment of the severity of depression and anxiety, the number of comorbidities, and the number of preventive actions and incidents/treatments undertaken after the event qualifying for the study.

Correlations	R	*p*
Assessment of depression severity and Number of comorbidities	1.323	0.186
Assessment of anxiety severity and Number of comorbidities	0.025	0.491
Assessment of depression severity and Number of preventive actions taken	−0.03	0.41
Assessment of anxiety severity and Number of preventive actions taken	0.2	0.841
Assessment of depression severity and Number of incidents/post-event procedures qualifying for the study	0.046	0.21
Assessment of anxiety severity and Number of incidents/treatments after the event qualifying for the study	1.262	0.578

Explanation of abbreviations: R—Spearman’s rank correlation value, *p*—the statistical significance.

**Table 7:** Characteristics of the study group, considering the correlation between gender and the number of preventive actions taken.

Correlations	Chi2	df	*p*
Gender and Number of Preventive Actions Taken	0.738	1	0.39

Explanation of abbreviations: Chi2—correlation value, df—number of degrees of freedom, *p*—the value of statistical significance.

**Table 8:** Multiple regression models—gender and severity of anxiety and depression.

Study Group (765; 100%)				
Variables	β	SE	t	*p*
**Assessment of anxiety severity**				
Coefficient	7.503	1.107	6.776	<0.001
Age	−0.016	0.016	−0.989	0.322
Sex—males	−1.703	0.306	−5.562	<0.001
Number of tertiary prevention activities implemented	−0.050	0.087	−0.568	0.570
Number of incidents and procedures	0.082	0.132	0.624	0.533
Number of comorbidities	0.140	0.119	1.178	0.239
**R:** 0.203 **R^2^:** 0.035 **F:** 6.477 ***p*-value:** <0.001 **EE:** 3.630				
**Assessment of depression severity**				
Coefficient	3.266	1.0128	3.22	<0.001
Age	0.027	0.014	1.882	0.060
Sex—males	−0.725	0.280	−2.590	0.010
Number of tertiary prevention activities implemented	−0.156	0.080	−1.945	0.052
Number of incidents and procedures	0.134	0.120	1.115	0.265
Number of comorbidities	0.201	0.109	1.847	0.065
**R:** 0.150 **R^2^:** 0.023 **F:** 3.478 ***p*-value:** <0.01 **EE:** 3.323				

Explanation of abbreviations: β—regression coefficient, SE—standard error, t—error quotient, *p*—statistical significance, R—correlation coefficient, R^2^—coefficient of determination, F—variance coefficient, EE—estimation error.

## Data Availability

Data available on request only for scientific purposes.
